# A Mobile App to Provide Evidence-Based Information About Crystal Methamphetamine (Ice) to the Community (Cracks in the Ice): Co-Design and Beta Testing

**DOI:** 10.2196/11107

**Published:** 2018-12-20

**Authors:** Louise Birrell, Hannah Deen, Katrina Elizabeth Champion, Nicola C Newton, Lexine A Stapinski, Frances Kay-Lambkin, Maree Teesson, Cath Chapman

**Affiliations:** 1 National Health and Medical Research Council Centre of Research Excellence in Mental Health and Substance Use National Drug and Alcohol Research Centre University of New South Wales Sydney Australia; 2 Department of Preventive Medicine Feinberg School of Medicine Northwestern University Chicago, IL United States; 3 Priority Research Centre for Brain and Mental Health The University of Newcastle Newcastle Australia

**Keywords:** internet, methamphetamine, mobile apps, mobile phone, substance-related disorder

## Abstract

**Background:**

Despite evidence of increasing harms and community concern related to the drug crystal methamphetamine (“ice”), there is a lack of easily accessible, evidence-based information for community members affected by its use, and to date, no evidence-based mobile apps have specifically focused on crystal methamphetamine.

**Objective:**

This study aims to describe the co-design and beta testing of a mobile app to provide evidence-based, up-to-date information about crystal methamphetamine to the general community.

**Methods:**

A mobile app about crystal methamphetamine was developed in 2017. The development process involved multiple stakeholders (n=12), including technology and drug and alcohol experts, researchers, app developers, a consumer expert with lived experience, and community members. Beta testing was conducted with Australian general community members (n=34), largely recruited by the Web through Facebook advertising. Participants were invited to use a beta version of the app and provide feedback about the content, visual appeal, usability, engagement, features, and functions. In addition, participants were asked about their perceptions of the app’s influence on awareness, understanding, and help-seeking behavior related to crystal methamphetamine, and about their knowledge about crystal methamphetamine before and after using the app.

**Results:**

The vast majority of participants reported the app was likely to increase awareness and understanding and encourage help-seeking. The app received positive ratings overall and was well received. Specifically, participants responded positively to the high-quality information provided, usability, and visual appeal. Areas suggested for improvement included reducing the amount of text, increasing engagement, removing a profile picture, and improving navigation through the addition of a “back” button. Suggested improvements were incorporated prior to the app’s public release. App use was associated with an increase in perceived knowledge about crystal methamphetamine; however, this result was not statistically significant.

**Conclusions:**

The *Cracks in the Ice* mobile app provides evidence-based information about the drug crystal methamphetamine for the general community. The app is regularly updated, available via the Web and offline, and was developed in collaboration with experts and end users. Initial results indicate that it is easy to use and acceptable to the target group.

## Introduction

In recent years, there has been widespread attention and concern across the globe as rates of methamphetamine production, consumption, and related-harms rise [1]. The crystalline form of methamphetamine, also known as crystal methamphetamine or “ice,” is typically the strongest and purest form of the stimulant drug. Crystal methamphetamine is now the main form of methamphetamine consumed and the number one drug of concern in Australia [2]. While population prevalence rates have remained relatively low and stable, with 6.3% of Australians aged over 14 years ever reporting using any form of methamphetamine [2], there is evidence that harms related to the use of crystal methamphetamine are increasing [3,4]. Data also indicate that rates of use in rural and regional areas of Australia are higher than that in metropolitan areas [5].

In response to the increasing harms and community concern about crystal methamphetamine, the Web-based *Cracks in the Ice Community Toolkit* [6] was developed and launched in April 2017. *Cracks in the Ice* is a freely available website funded by the Australian Government Department of Health to provide trusted, evidence-based, and up-to-date information about crystal methamphetamine for the Australian community. The website was developed in collaboration with the community, leading experts in the field, and consumer experts, over an 18-month period from 2015 to 2016. The development was a broad-reaching and iterative process (for further details about the development process and beta testing see Ref. [7]). Its target audience includes people who use crystal methamphetamine, their friends and family, health care professionals, schools, and general community members with an interest or concern about the drug. The website includes resources that were developed by the research team in relation to the most up-to-date evidence and external resources. Prior to inclusion on the website, external resources (including fact sheets, guidelines, and Web-based programs) were independently reviewed by the *Cracks in the Ice* project team. Resources were assessed for eligibility for inclusion using an adapted version of the National Health and Medical Research Council (NHMRC) Body of Evidence Matrix (2009; see Multimedia Appendix 1) against the following criteria: evidence base, impact and utility, generalizability, applicability (applicable to an Australian context), recent (resource updated in the past 10 years), and duplication. Textbox 1 summarizes the website content. The website is regularly monitored, and website traffic tracked through Google Analytics. To ensure the information and resources remain up-to-date and include the latest evidence, the website content is reviewed on a regular basis with a systematic review of all content conducted once per year. Since launching in April 2017, the website has reached >79,000 unique users (as of May 2018). While the website usage has shown steady growth since launch, the website was primarily designed to be viewed on a desktop or laptop computer. The *Cracks in the Ice* app aims to bring together the best available evidence about crystal methamphetamine and improve access for the community to accurate information, including information about the effects of ice, where or how to seek help, and relevant support services. The provision of accurate information is an important part of community prevention strategies. While *Cracks in the Ice* is not a treatment intervention, it aims to promote help seeking by providing up-to-date, accurate information about treatment options, service contact details, and conversation starters. Furthermore, community consultation during the development of the Web-based toolkit indicated that Australian community members were seeking evidenced-based information about ice [7]. However, evidence indicates that the quality of information currently available on this topic in an app-based format is poor, and no existing apps focusing on crystal methamphetamine have undergone evaluation [8].

Recent data indicate that people are increasingly using mobile devices to access the internet, with mobile devices now the most frequently used device to access the internet in Australia [9]. Furthermore, consultation with community members, as part of the development process of the *Cracks in the Ice* website, indicated that nearly two-thirds (287/451, 63.6%) of participants said that they would use a mobile device to access an information website about crystal methamphetamine.

Over the past decade, there has been a proliferation of smartphone device ownership, particularly in high-income countries. The smartphone adaption in the United Kingdom rose from 52% of the population in 2012 to 85% in 2017 [10]. Australians have some of the highest smartphone ownership in the world, with 88% of the Australian population owning a smartphone in 2017, up from 84% in 2016 [11]. Mobile apps can extend the reach of public health information and offer offline capabilities, improving access for rural and regional communities, where access to the internet may be unreliable.

A recent review by our team of mobile apps containing information about methamphetamines, including crystal methamphetamine, identified a clear shortage of high-quality and engaging apps providing educational information [8]. To address this gap, a companion *Cracks in the Ice* mobile app was developed. The target audience and anticipated end users for the app mirrored that of the *Cracks in the Ice* website, including people who use crystal methamphetamine, their friends and family, health care professionals, schools, and general community members with an interest in, or concern about, the drug.

**Key content areas of the *Cracks in the Ice* website and mobile app.**

**Get the facts about ice**

- What is ice
- How many people use ice
- What are the laws about ice

**Staying safe**

- When and where to get help (key support services in Australia)
- How to support a loved one
- Protecting yourself and others
- Support for Aboriginal and Torres Strait Islanders

**What are the effects of ice?**

- How ice works in the brain and body
- The mental health effects of ice
- Using ice with other drugs

**Tailored resources for specific groups**

- Community groups
- Families and friends of people who use ice
- Schools
- Health professionals

This paper describes the development and beta testing of the *Cracks in the Ice* mobile app, the first of its kind to provide evidence-based information about crystal methamphetamine in a mobile app format. The app aims to extend the reach and dissemination of high-quality information about crystal methamphetamine in Australia through an easily accessible and engaging mobile format. Specifically, the app has been designed to provide a condensed, offline-accessible version of the *Cracks in the Ice* Web-based toolkit [6], tailored to smartphone and tablet devices. These offline capabilities extend previous dissemination efforts by improving access for those without reliable internet access such as rural and regional residents. As with the website, the app has been designed to increase access to information about crystal methamphetamine, rather than actively facilitating behavioral change among end users (such as monitoring or decreasing drug use). The objective of the beta testing was to trial the *Cracks in the Ice* app with general community members, focusing on the usability, functionality, design, visual appeal, and app engagement. Furthermore, beta testing aimed to explore the impact of the app on perceived knowledge about crystal methamphetamine, attitudes toward crystal methamphetamine, and help-seeking intentions.

## Methods

### Overview of the Co-design Process

The *Cracks in the Ice* mobile app was developed over 4 months through a collaborative and iterative process using co-design. Importantly, co-design includes end users (people who will use the app after development) in the design process as experts on their experiences [12]. The inclusion of end users into the development of initial design concepts has been shown to result in outcomes with greater benefit to the user than ideas generated by in-house experts alone [13]. An established Expert Advisory Group (EAG), consisting of leading experts in drug and alcohol prevention and treatment, internet interventions, and mobile app development, provided guidance and recommendations throughout the development process. Multiple stakeholders were consulted throughout the process, including the core research team (that has expertise in addiction and mental health and was responsible for project management and oversight), app developers, a consumer expert with lived experience of addiction, and a sample of end users from the Australian community. Consumer participation was incorporated to ensure perspectives and needs of those directly impacted by the research were considered. Consumer participation is considered important for optimizing research outcomes across health fields, including mental health [14,15] and drug treatment [16].

**Figure 1:**
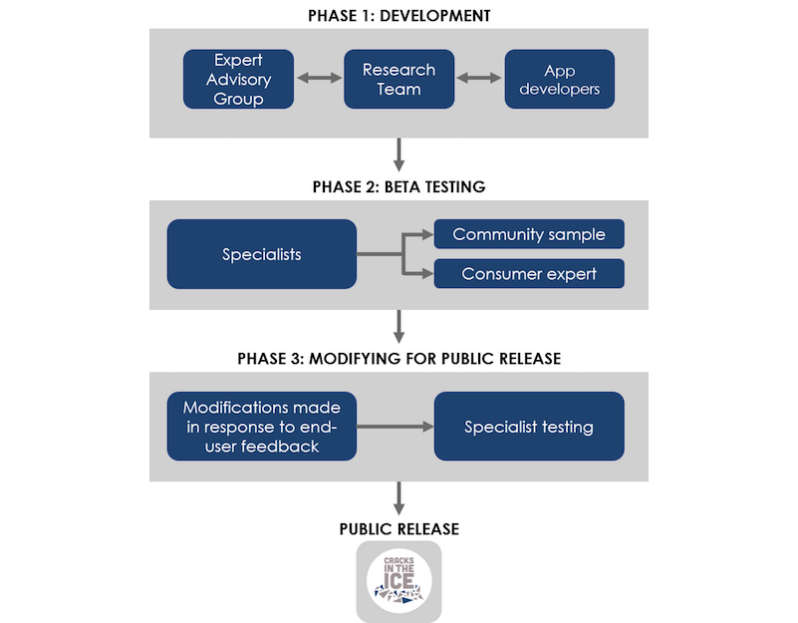
The co-design process.

The co-design process (Figure 1) consisted of 3 phases as follows:

1. Development of a beta version of the app, in collaboration with app developers and consultation with the EAG.
2. Beta testing among the EAG, app developers, and a community sample of end users (members of the Australian general population, including people who use crystal methamphetamine, their families and friends, health professionals, and concerned members of the community), and a consumer expert with lived experience of addiction.
3. Modifications to the app in response to end user feedback prior to public release.

### Phase 1: Development of the Beta Version of the *Cracks in the Ice* App

A beta version of the app was collaboratively developed by the research team, app developers, and EAG. To optimize reach, the app was designed to be compatible with the 2 most popular operating systems—iOS and Android. All app content was sourced from the existing Web-based toolkit [6] and included general information about crystal methamphetamine, its physical and mental health effects, and how to access support and treatment services, as well as more targeted resources for families and friends of individuals affected by crystal methamphetamine, health professionals, school, and community groups.

**Figure 2:**
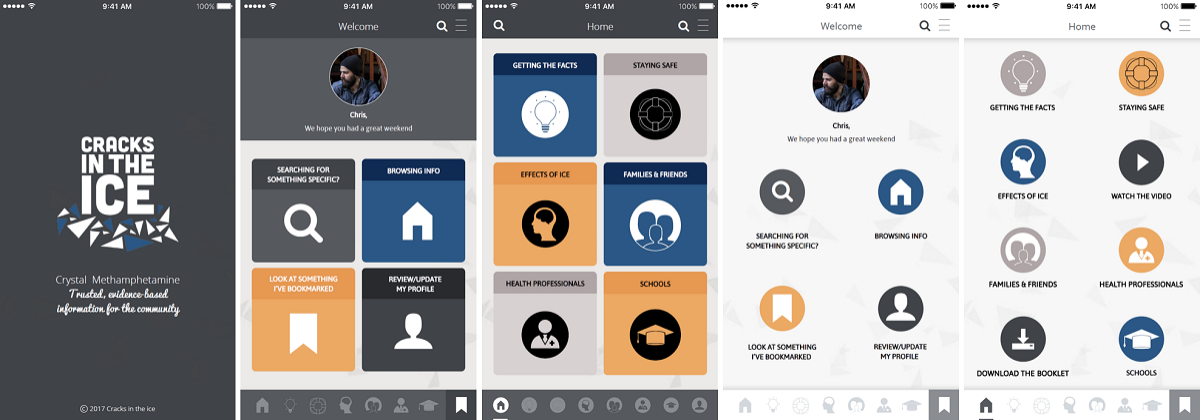
Example preliminary design options.

The app’s structure and overall visual design (ie, branding, color scheme, and font) were designed to be consistent with the Web-based toolkit, which was informed by consultations and feedback with end users [7]. Figure 2 presents screenshots of the app’s preliminary design options. Several functions from the toolkit were built into the app, including the following: (1) a search bar, allowing users to search for specific information by keyword(s); (2) a bookmarking function, allowing users to log in, save, and revisit specific sections or resources; and (3) a share button, allowing users to share content with others through email or social media. Two new functions included offline capabilities, allowing access to key information and resources without an internet connection, and “push notifications,” allowing users to keep up-to-date with new resources, as they become available.

To ensure the toolkit’s Web-based content was ready for transfer to mobile, all webpages from the toolkit were assessed for length and text density by 2 members of the research team. Pages considered too text heavy for mobile display were then condensed. To ensure no important information was lost during this process, each condensed page was then compared with the original Web-based version by another independent reviewer.

The content was then optimized for mobile display. Specifically, the Web-based content was consolidated into blocks of content known as snippets. Snippets are pieces of dynamic content (text, images, etc) that can be styled independently from the rest of the page and are often used to make large sections of text easier to read and navigate on mobile display. In addition, the Web content was packaged in the show/hide or “accordion” style sections in the app. Show/hide sections are responsive displays that expand and retract when the end user taps on them. When tapped by users, the section expands to “show” the full piece of content (eg, the full paragraph of text). Snippets and show/hide sections were designed to work together to enhance clarity and lessen the amount of scrolling for end users.

### Phase 2: Beta Testing

At the completion of phase 1, the first beta version of the app was scripted in both iOS and Android operating systems for testing. As the beta testing study was a pilot, with the purpose of improving the app prior to release, it focused on the acceptability, usability, perceived knowledge, and attitudes after using the app. Beta testing consisted of 2 phases as follows: (1) specialist testing; and (2) end user testing. Specialist testing involved 4 experienced app developers and 2 members of the research team running initial tests to check for software bugs, errors, and crashes. A revised version of the app was then sent out for end user testing among a community sample and an expert consultant with lived experience of addiction.

#### Design and Procedure

End user beta testing took place over a 1-week period during September 2017. All aspects of this study were approved by the University of New South Wales Human Research Ethics Committee (HC15732). Testing was conducted through an anonymous internet survey (of approximately 30 minutes; a full copy of the survey is available on request). Participants were recruited through paid Facebook advertising, as well as through electronic notifications sent to the existing *Cracks in the Ice* website subscriber list and posted on the *Cracks in the Ice* Facebook and Twitter pages. Paid Facebook advertising was broadly targeted at Australian community members over the age of 16 years. Facebook recruitment for health research has been shown to result in samples that are generally representative of the total population and is particularly useful in engaging hard-to-reach populations, such as people who use drugs, while traditional methods tend to underrepresent these groups [17].

The survey was open to Australian residents aged ≥16 years who had access to an iOS or Android device capable of downloading and running mobile apps. All respondents were required to provide informed consent and were given the opportunity to enter a draw to win an iPad at the completion of the survey as reimbursement for their time.

On starting the survey, all participants were asked to download and preview the beta version of the app for 5-10 minutes before answering questions about its functionality, visual appeal, usability, and engagement. To ensure respondents used the app for a minimum of 5 minutes before providing feedback, a timer was incorporated into the survey preventing respondents from completing the evaluation questions until at least 5 minutes had passed.

#### Measures

The demographic data collected included gender, age, state or territory of residence, Aboriginal or Torres Strait Islander heritage, occupation, and number of children. In addition, participants were asked whether they had ever used the drug ice (yes or no), their frequency of use in the past year (ranging from “never” to “once a week”), whether they knew someone who uses the drug ice (yes or no), and whether they knew a friend or family member who uses ice (yes or no). Furthermore, participants were asked to report if they used a mobile or tablet device to access the app, how much prior experience they had of using mobile apps, whether they were aware of the *Cracks in the Ice* Web-based toolkit (yes or no), and how familiar they were with the Web-based toolkit prior to using the app.

Respondents were asked to rate the overall appeal of the app, layout, visual design, ease of use, features, and functions of the app through questions such as, “what do you think about the overall visual design of the app?” (rated from 1 “strongly dislike” to 5 “strongly like”). Several of these questions were adapted from the Mobile Application Rating Scale (MARS) [18], including questions measuring the likelihood to recommend the app to others, or use the app in future, as well as 2 questions assessing how easy and interesting the app was to use. The MARS is a well-established rating scale used by both professionals and end users to assess the quality of mobile apps. In addition, respondents were given the opportunity through open-ended questions to provide any suggestions for improvements or suggest additional content or features that they thought should be included.

To assess whether the app had any impact on an end user’s self-reported knowledge of crystal methamphetamine, respondents’ perceived level of knowledge about crystal methamphetamine was assessed before and after using the app. Specifically, participants were asked to rate their perceived knowledge of the drug crystal methamphetamine on a 4-item Likert scale ranging from 0 (“I have no knowledge of the drug ice”) to 4 (“I am very knowledgeable about the drug ice”). Additional questions, adapted from the MARS, assessed whether respondents believed the app would have any impact on awareness, knowledge, and understanding of crystal methamphetamine or crystal methamphetamine prevention messages, attitudes toward crystal methamphetamine use, actual crystal methamphetamine use, and help-seeking behaviors among others who use the app.

#### Data Analysis

Data analyses were conducted in IBM SPSS Statistics 24 [19]; this included descriptive statistics and a Wilcoxon signed-rank test to investigate a change in participants’ perceived level of knowledge about crystal methamphetamine after they had used the app.

### Phase 3: Modifying the App for Public Release

Revisions were made in response to end user feedback to improve the app before its public release. Another round of testing by the research team ensured all changes were implemented correctly.

## Results

### Phase 1: Development of the Beta Version

**Figure 3:**
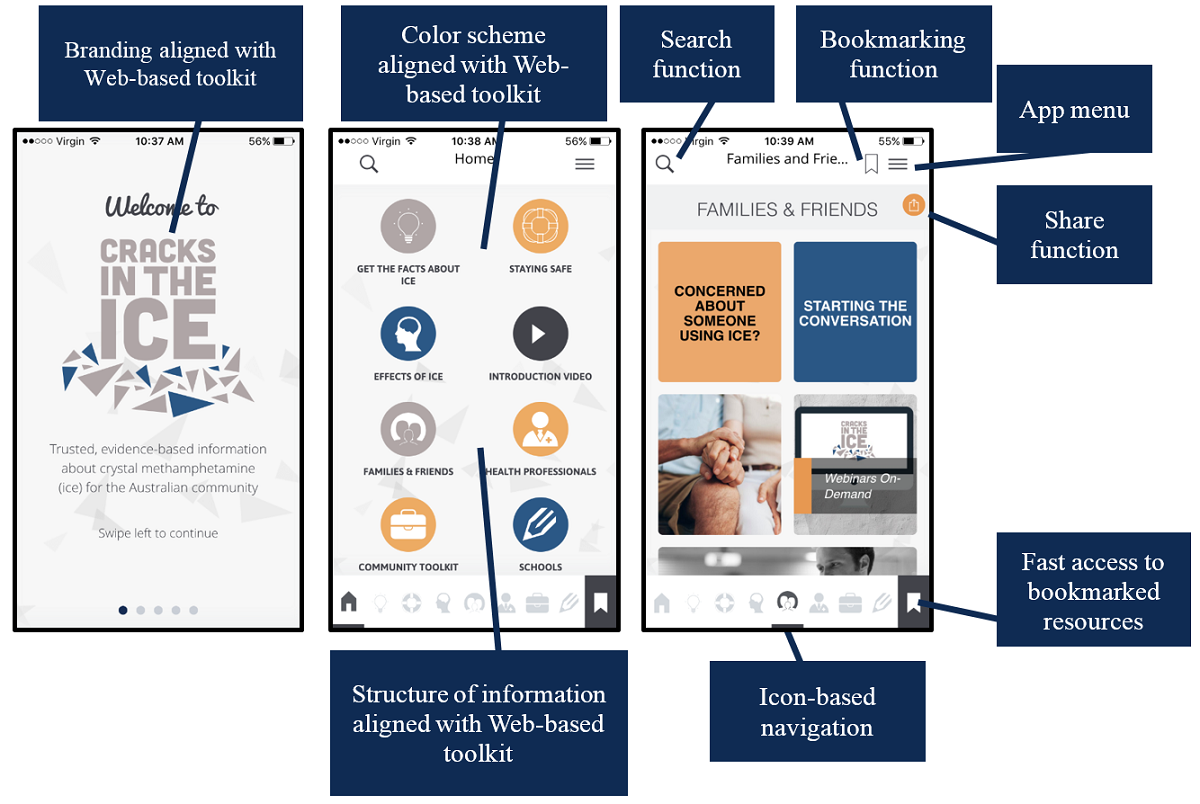
Key design elements and features of the beta version of the *Cracks in the Ice* mobile app.

Figure 3 summarizes key design elements and features of the final beta version of the *Cracks in the Ice* mobile app.

### Phase 2: Beta Testing

#### End User Survey

##### Participants

A total of 34 participants completed the survey [age range, 21-60 years; mean, 37.2 (SD 10.1) years]. Table 1 summarizes further descriptive statistics.

Nearly all participants (33/34, 97%) were aware of the drug crystal methamphetamine (“ice”), with 82% (28/34) reporting they had some knowledge of the drug or were very knowledgeable about the drug. Furthermore, the majority of participants (24/34, 71%) were aware of the *Cracks in the Ice* Web-based toolkit.

##### Overall Response to the App

Participants’ overall response to the app was positive. The app received a high average star rating of 3.8 out of 5, where a score of 1 corresponds to “one of the worst apps I’ve used” and 5 corresponds to “one of the best apps I’ve used.” Most participants (28/34, 82%) “liked” or “strongly liked” the app overall. In addition, 94% (32/34) said that they would use the app again in the next 12 months if it was relevant to them, and 65% (22/34) said that they would recommend the app to “many people” or “everyone” who might benefit from using it. Table 2 provides examples of qualitative feedback.

**Table 1:** Sample characteristics of survey respondents.

Characteristics			n (%)
**Sex**			
	Male	7 (21)	
	Female	27 (79)	
**Aboriginal or Torres Strait Islander**			
	Yes	3 (9)	
	No	31 (91)	
**State of residence**			
	New South Wales	16 (47)	
	Queensland	6 (18)	
	South Australia	5 (15)	
	Victoria	2 (6)	
	Western Australia	2 (6)	
	Northern Territory	1 (3)	
	Tasmania	1 (3)	
	Australian Capital Territory	1 (3)	
**Area**			
	Metropolitan area	19 (56)	
	Regional area	9 (26)	
	Rural area	6 (18)	
**Employment**			
	Employed	32 (94)	
	Unemployed	2 (6)	
**Employment type**			
	Health Professional	13 (38)	
	Professional worker	9 (26)	
	Student	6 (18)	
	Other	6 (18)	
**Ever used the drug ice**			
	Yes	6 (18)	
	No	28 (82)	
**Used ice in the past year**			
	Yes	1 (3)	
	No	33 (97)	
**Know a friend who uses ice**			
	Yes	14 (58)	
	No	10 (42)	
**Have a family member who uses ice**			
	Yes	8 (33)	
	No	16 (67)	
**Mobile platform used to test app**			
	iPhone	20 (59)	
	Android	14 (41)	
**Previously downloaded an app**			
	Yes	33 (97)	
	No	1 (3)	
**Use apps several times a week**			
	Yes	34 (100)	
	No	0 (0)	
**Downloaded an app in the last week**			
	Yes	24 (71)	
	No	10 (29)	
**Typically use apps several times a day**			
	Yes	24 (71)	
	No	10 (29)	

**Table 2:** Example feedback from end users about the beta version of the app.

Type of feedback and app feature		Example feedback
**Positive feedback**		
	Information and resources	“I like the fact that there are resources on the app that are proven and reliable, and the way the information is presented is attractive and interesting” “I like the app because it is easy to use, provides evidence-based information. Also, the fact that there is up to date information for health professionals as well.” “Caters to different groups/stakeholders”
	Ease of use and navigation	“It's well designed and easy to use” “I love that it is almost a mirror of the website, so it will make it easier to use in my work.” “Smooth UI/UX^a^”
	Visual design	“Beautiful design” “I love the colour scheme of the app. It's warm and inviting without being over the top which may be distracting.” “I really love the small icons at the bottom of screen.”
**Negative feedback**		
	Too much information	“Too much information—overwhelmed me” “Lot of information”
	Text heavy	“It’s not bad but very wordy.” “I thought it was kind of wordy in places.”
	Low engagement	“Bit boring”
	Too similar to the website	“It also comes off as a pure copy of the website rather than something new.” “Seemed very similar to the website”

^a^UI/UX: user interface/user experience.

**Table 3:** The frequency and proportion of respondents endorsing knowledge items before and after using app (n=34).

Response items	Before using app, n (%)	After using app, n (%)
I have no knowledge about the drug ice	2 (6)	0 (0)
I know very little about the drug ice	4 (12)	0 (0)
I have some knowledge about the drug ice	10 (29)	15 (44)
I am very knowledgeable about the drug ice	18 (53)	19 (56)

**Table 4:** Agreement with statements regarding app’s impact on awareness, knowledge, understanding, attitudes, motivation, and behavior.

Statement	Strongly disagree, n (%)	Disagree, n (%)	Neutral, n (%)	Agree, n (%)	Strongly agree, n (%)
This app is likely to increase awareness of ice/ice prevention messages	0 (0)	5 (15)	4 (12)	17 (50)	8 (24)
This app is likely to increase knowledge and understanding of ice/ice prevention messages	0 (0)	2 (6)	6 (18)	18 (53)	8 (24)
This app is likely to change attitudes toward ice use	0 (0)	8 (24)	9 (26)	15 (44)	2 (6)
This app is likely to increase motivation to reduce ice use	2 (6)	6 (18)	17 (50)	7 (21)	2 (6)
Use of this app is likely to encourage further help seeking for ice use (if it’s required)	1 (3)	1 (3)	10 (29)	19 (56)	3 (9)

##### Feedback on Specific App Features

Most participants (28/34, 82%) reported that they “liked” or “strongly liked” the overall visual design and layout of the app, with the majority (28/34, 82%) reporting the images and infographics were engaging. Most participants (29/34, 85%) found the app to be “moderately” or “very” interesting to use. The usability rated highly. Almost half the sample (15/34, 44%) reported being “able to use the app immediately,” and over a third (12/34, 35%) found the app “easy to learn how to use” or agreed it “had clear instructions.” Almost all participants agreed that offline functionality (32/34, 94%) and automatic information updates (33/34, 97%) would be useful.

##### Perceived Change in Personal Knowledge of Crystal Methamphetamine

A high number of participants indicated that they had a moderate-to-high level of knowledge about crystal methamphetamine after using the app, compared with prior to using the app (see Table 3). While we observed a trend toward increased perceived knowledge about crystal methamphetamine after using the app, this difference was not statistically significant (*z*=−1.90, *P*=.058).

##### Perceived Impact on Others’ Awareness, Knowledge, Understanding, Attitudes, Motivation, and Behavior

Table 4 presents descriptive statistics in relation to the apps perceived impact on others’ knowledge, attitudes, and behavior. The majority of participants “agreed” or “strongly agreed” that the app would likely increase awareness, knowledge, and understanding of crystal methamphetamine and associated prevention messages, as well as encourage further help seeking. Half “agreed” or “strongly agreed” the app would likely change attitudes toward crystal methamphetamine use. Comparatively fewer participants agreed the app would increase motivation to reduce crystal methamphetamine use.

##### Suggested Improvements

When asked about potential improvements to the app, almost all participants (32/34, 94%) agreed that they would like to be able to control how frequently the app sends notifications. Options to personalize other app features, such as the color scheme, font size, font style, welcome message, and password log-in, were less popular, with <30% endorsing each of these suggested changes. Almost two-thirds (22/34, 65%) wanted to see more infographics and images incorporated.

Suggestions for improvement were submitted through open-ended feedback and the most commonly cited included adding more information for people who use crystal methamphetamine, incorporating more information about national and local support services, and improving navigation. Other suggestions included making the app more interactive, incorporating stories of lived experience, and tailoring the information to be more mobile friendly. Multimedia Appendix 2 provides example qualitative feedback. When participants were asked how important it was for the app developers to action their suggested improvements, 35% (12/34) classified their suggested changes as “important,” with the remaining participants being “unsure” (10/34, 29%) or classifying their suggestions as “not necessary/just a suggestion (the app would work well or very well without these improvements)” (12/34, 35%).

#### Consumer Expert Feedback

Feedback from the consumer expert was largely in line with the community sample. The expert “liked” the app overall, rating it 4 out of 5 stars, reporting they would recommend it to “many people.” It was noted that the app was easy to navigate through its icon-based design. As with the community sample, the overall visual design, layout, engagement, functionality, and usability of the app was rated highly, and offline functionality and updates were endorsed as useful features.

The expert “strongly agreed” the app is likely to increase people’s awareness, knowledge, and understanding of crystal methamphetamine and crystal methamphetamine prevention messages, and encourage further help seeking. They “agreed” it would likely change attitudes toward crystal methamphetamine use and increase motivation to reduce use. It was suggested that more images and infographics would improve the app, as well as more quotes from people with lived experience. In addition, it was suggested that the option for users to upload a picture to their profile should be removed, to eliminate the possibility that app users’ identities could be exposed when viewing and bookmarking sensitive information about drug use.

### Phase 3: Modifying the App for Public Release

#### Summary of Modifications

**Figure 4:**
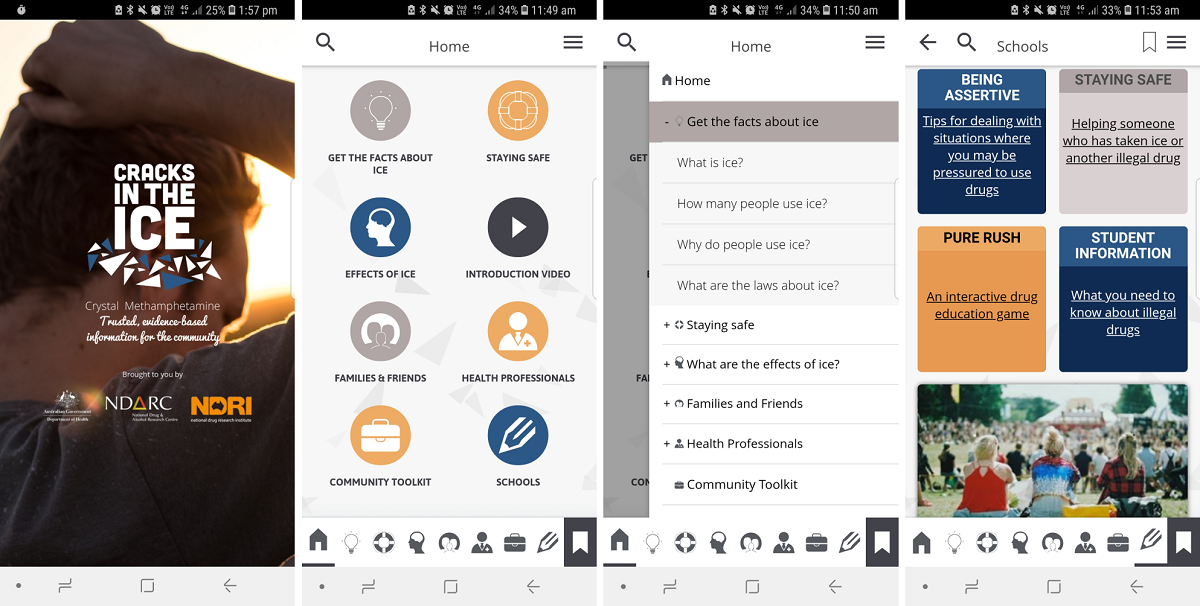
Screenshots of Cracks in the Ice mobile app when released to the public January 2018. Source: Cracks in the Ice App Version 1. Developers: National Health and Medical Research Council Centre of Research Excellence in Mental Health and Substance Use and Netfront Pty Ltd. Licensed under fair use.

Revisions were made in response to end user and expert feedback, and these are summarized in Table 5. Figure 4 provides screenshots of the final app released to the public.

### Usage Statistics

The *Cracks in the Ice* mobile app was officially launched on January 9, 2018. Approximately 4 months following launch, 797 unique users had downloaded the app (measured by “installs by user” on Google Play and “app units” on iTunes). Downloads have primarily been to devices within Australia (571 downloads); however, there has also been some interest from the United States (132 downloads). Uninstalls monitored through the Google Play store indicated a substantial proportion of Android app users (57.8%) have uninstalled the app after download. To investigate potential reasons for uninstalls, an optional open feedback form has since been incorporated in both the Android and iTunes app. Users are given the option of providing their feedback through this form within the first 10 minutes of app use and again one week later.

**Table 5:** Summary of feedback and modifications made to the app.

Suggestions from feedback	Modification(s) made
Include more images and infographics^a^	More show/hide displays, images and infographics were incorporated to make the app’s content more mobile friendly. The majority of existing infographics were also optimized for mobile display.
Improve navigation^a^	To improve navigation, a back button was added to each of the 6 information tabs. This new feature allows users to easily navigate back to pages recently visited and return to the home page.
Improve navigation^a^	To further assist end users using the app for the first time, more references to the app’s icons (corresponding with the app’s 6 information tabs) were incorporated to ease navigation.
Add more information for people who use crystal methamphetamine, incorporating more national and local support services^b^	The listing of National Support Services was reviewed and considered sufficient. A local support list and inclusion of more information for people who use crystal methamphetamine was not implemented as it was considered outside of the scope of the initial release. The suggestion will be considered in later releases.
Potential for people’s identities to be exposed when viewing and bookmarking sensitive information about drug use^a^	In accordance with the consumer expert’s feedback, the profile picture function was disabled in the final version of the app.
Technical bugs^a^	All technical bugs were resolved and retested.

^a^Feedback actioned prior to the final release.

^b^Feedback not actioned

## Discussion

### Principal Results and Comparison With Prior Work

To date, there has been a lack of easily accessible, up-to-date, evidence-based information about the drug crystal methamphetamine (“ice”) for the general community. This is of particular importance in Australia, where community concern about crystal methamphetamine is at an all-time high [2], and harms related to crystal methamphetamine are rising [3,4]. This paper described the co-design and beta testing of the first mobile app, developed in collaboration with end users (Australian general community members) and leading experts, to provide easy access to evidence-based information and support options for crystal methamphetamine for the general community. The app was based on the Web-based *Cracks in the Ice Community Toolkit* [6] and provides an alternative method for community members to access information about crystal methamphetamine, in a convenient and engaging format.

Results indicated that the app was very well received and strongly liked. In particular, participants responded positively to the evidence-based information provided, usability, and visual appeal. Previous reviews have found that available drug-related mobile apps are rarely supported or developed with research evidence [20,21], with some apps even promoting illicit drug use through simulated drug taking and dealing [22]. Within this landscape, there is a clear need for accurate public health information and it is encouraging that the evidence base of the information was very positively rated by end users (Australian general community members). Yet, it is equally important that mobile apps are easy to navigate and engaging for users. Areas suggested for improvement in the current app included reducing the amount of text, increasing engagement, removing a profile picture, and improving navigation through the addition of a “back” button; all these suggestions were incorporated in the final version prior to public release, and it is likely that improvements to app engagement will increase usage and enhance recall of information. The revisions made to increase engagement and visual appeal make the *Cracks in the Ice* app the first of its kind to present evidence-based information in an engaging and appealing way to end users. Removal of the option to add a profile picture is in line with recommendations from a review of the potential of smartphones in addiction research and treatment, which outlines that protecting user privacy is a key ethical consideration when developing apps [23]. Although the current app does not actively collect information about drug taking or illegal behavior, it does focus on an illicit drug and users may prefer to remain anonymous.

While a higher number of participants agreed they had some knowledge of crystal methamphetamine or were very knowledgeable about the drug after using the app (compared with prior to using the app), this difference did not reach statistical significance; this is most likely because of the high baseline knowledge of the sample, with 82% (28/34) reporting they had at least some knowledge about crystal methamphetamine prior to using the app, therefore making it harder to see knowledge changes because of ceiling effects. While marked knowledge effects were not found in this study, participants did agree the app was likely to increase awareness, knowledge, and understanding about crystal methamphetamine. It is promising that most participants agreed the app would encourage help seeking; however, only one-quarter agreed the app would increase motivations to decrease actual crystal methamphetamine use. This is consistent with the app’s goal, to provide high-quality, evidence-based information, rather than affect behavioral change.

While some end users offered suggestions to include more information about national and local support services, such directories require regular maintenance to avoid becoming out of date. In reviewing this request, it was decided the existing list of National Support Services, including >12 national directories and specific services for Aboriginal and Torres Strait Islander people, was sufficient. Listings of local services were considered outside of the scope of the current project to maintain. In addition, suggestions to add more information targeted at people who use crystal methamphetamine and include more stories of lived experience were considered valuable but also outside of the scope of modifications for the first release of the app; these suggestions will be considered for future releases.

Several findings merit further discussion. Notably, nearly all participants rated the app’s ability to function while offline (once downloaded) and provide automatic updates to content, as useful. These 2 functions are relatively unique, setting the current app apart from others in the health information landscape. Offline availability is emerging as an important function for apps delivering time-critical information, for example, in clinical and disaster information, in which intermittent internet access can have detrimental effects [24,25]. Offline capabilities were built in the current app as a core function to enable access to key information and resources for people in areas where internet access may be unreliable (ie, rural or regional areas). This is particularly valuable in Australia, where there is evidence that the use of crystal methamphetamine is higher in remote rural areas [5], where internet connections are typically less reliable than that in urban areas. In addition, the app was built to automatically update content when updates are made to the companion website. The *Cracks in the Ice* website content is systematically reviewed once a year, with ad-hoc updates made as needed, to ensure new evidence and resources are included as they become available. Moreover, this process includes the removal of inactive or out-of-date links and resources as necessary. Such systematic maintenance is a unique feature in the quickly changing landscape of mobile apps and health information, and ensures the *Cracks in the Ice* app will remain current and based on the latest scientific evidence.

### Strengths and Limitations

The key limitation of this study was that the beta testing was a pilot study of a small group of community members exploring the acceptability, usability, attitudes, and knowledge pre- and postapp usage. The small number of participants may not reflect the diverse Australian population. Females were overrepresented in the sample (27/34, 79% females), and a significant number of participants were based in one Australian state (16/34, 47%); however, the study did include at least 1 participant from each state and territory in Australia. It should be noted that a relatively high number of participants (24/34, 71%) were aware of the *Cracks in the Ice* Web-based toolkit; this is likely attributed to the promotion of the beta testing survey through existing networks associated with *Cracks in the Ice*, including an associated Facebook page and Twitter account. While this group represents a key target audience, a future challenge may be how to engage and attract others, who are unfamiliar with the Web-based toolkit, to use the app. As this was a feasibility and acceptability study, it is not possible to make conclusions about the app’s effects on knowledge, drug usage, and help-seeking behavior. A larger randomized controlled design with larger sample size is needed to test the app’s effects on these constructs. A further limitation of this study is the short length of time participants were asked to use the app during beta testing. While the beta testing period was short, the development and expert testing by researchers and Web developers involved an in-depth examination of all content and design features over a period of months, so that the entire content was examined in-depth during the design process overall. While valuable information would have been gained by asking participants to go through the app in-depth over a period of several weeks, this would not have represented how most people use mobile apps in a real-world context. Furthermore, the aim of beta testing was to gain feedback on the feasibility and initial acceptability of the app from people who are likely to use the app after public release. It is the standard practice in the field to gain app evaluations after using an app for a short period (see MARS recommendations to use an app for a minimum of 10 minutes; Stoyanov et al [18]).

Key strengths of this study include the co-design approach, automated updating of the information on the app, the ability to access the app on both the iPhone and Android operating systems, and the availability of information offline once the app is downloaded. Another key strength was the inclusion of people with lived experience of the drug crystal methamphetamine. A substantial portion of the sample reported they had used crystal methamphetamine (6/34, 18%), and over half reported they had a friend who used crystal methamphetamine, with one-third reporting they had a family member who used crystal methamphetamine. These figures are much higher than national averages and give confidence that the target audience (people with experience or interest in the drug crystal methamphetamine) were included in the app design and testing. While the focus of this study was on the development and beta testing of the app, it will be of interest to track the app usage and explore how different end users engage with the app. It is envisaged that people will use the app in different ways depending on their circumstances, for example, rural health professionals interacting with clients who use drugs may utilize the offline capability repeatedly, while a family member wanting support services for their loved one who is at the point of crisis may find the resources they need in a single visit.

Although mobile apps with some focus on crystal methamphetamine do exist, for example, drug handbooks, games, and apps tracking drug use, very few have been developed through a scientific process or have involved co-designing with key stakeholders [8]. The development of the *Cracks in the Ice* mobile app used a co-design process, involving experts, researchers, app developers, a consumer expert with lived experience, and beta testing among a sample of app end users from the Australian community; this resulted in a wide range of perspectives being incorporated into the app, ensuring the final product was grounded in scientific evidence, and useful, relevant, and engaging for end users.

### Conclusions

This is the first study to describe the co-design and beta testing of a mobile app to disseminate evidence-based information about the drug crystal methamphetamine or “ice.” The design was an iterative process, incorporating a number of key stakeholders, including public health experts, app developers, end users, and a consumer expert. Initial findings show the app was well received and rated highly in terms of the usability, design, and the provision of high-quality information. Key improvements to the app included the addition of more infographics and images, show/hide test displays, and the addition of a back button to assist with app navigation. In addition, initial results indicate the *Cracks in the Ice* mobile app is easy to use, engaging, and acceptable to the target group. Translation of evidence-based information into a mobile app format, accessible offline, has the potential to increase the reach and impact of information and support services for people impacted by crystal methamphetamine.

## Appendix

Multimedia Appendix 1 Adapted version of the National Health and Medical Research Council Body of Evidence Matrix.

Multimedia Appendix 2 Example suggestions for improvement from end users about the app beta version.

## Abbreviations

EAG: Expert Advisory Group
MARS: Mobile Application Rating Scale
NHMRC: National Health and Medical Research Council
