# Supplementary material for: A Mobile App to Provide Evidence-Based Information About Crystal Methamphetamine (Ice) to the Community (Cracks in the Ice): Co-Design and Beta Testing
Source: JMIR Mhealth Uhealth. 2018 Dec 20;6(12):e11107. doi: 10.2196/11107 (PMC6320422; doi:10.2196/11107)
Supplement: Multimedia Appendix 1 [file mhealth_v6i12e11107_app1.pdf]

Multimedia Appendix 1

Adapted version of the National Health and Medical Research Council Body of Evidence Matrix.

Component	A	B	C	D
	**Excellent**	**Good**	**Satisfactory**	**Poor**
**Evidence base:** was the resource developed on the basis of evidence?	Formally evaluated and findings published.	No formal evaluations; Developed on the basis of published findings OR some testing among end users has been conducted.	No reference to formal evidence or testing; developed with formal input from experts.	Developed on basis of personal opinion only/unknown.
**Impact and utility:** range and importance of issues covered	**Very large**: covers a range of relevant issues comprehensively.	**Substantial**: covers more than one relevant issue.	**Moderate**: covers a single issue of high importance.	**Slight or restricted**: brief resource, covers a single issue of lesser importance.
**Generalizability:** relevance of the resource to the community and/or target groups for the toolkit	Relevant to one or more of the toolkit's target groups.			Not relevant for any of the toolkit's target groups.
**Applicability:** how applicable is the resource to an Australian context?	Directly applicable to the Australian context.	Applicable to Australian context with some caveats.		Not applicable to the Australian health care context.

External resources were assessed according to the criteria outlined above and using the following steps:

- Prior to the formal assessment process, three resources were rated by all project team members, and ratings were discussed to ensure the validity of the scale for this purpose.
- Resources needed to score a C (a satisfactory rating) or above on all four components of the scale, except in exceptional circumstances (eg, a rating of D for impact and utility).

- Each resource was reviewed by one member of the team. If the resource did not receive a score that warranted inclusion, the resource was discussed by team at a team meeting, and if consensus could not be reached, expert opinion was sought. Only one resource was deemed ineligible for inclusion on the toolkit and was excluded owing to considerable overlap in content with another, more recently published, resource.
