# Supplementary material for: A Mobile App to Provide Evidence-Based Information About Crystal Methamphetamine (Ice) to the Community (Cracks in the Ice): Co-Design and Beta Testing
Source: JMIR Mhealth Uhealth. 2018 Dec 20;6(12):e11107. doi: 10.2196/11107 (PMC6320422; doi:10.2196/11107)
Supplement: Multimedia Appendix 2 [file mhealth_v6i12e11107_app2.pdf]

Multimedia Appendix 2

Example suggestions for improvement from end users about the app beta version

Area of improvement	Example feedback
Information for ice users	*"Some more fundamental and specific harm reduction tips regarding danger from pipes, how to prevent spreading blood borne diseases, and vein care."*<br>*"Strategies for substance users to make changes - delay, distract etc"*
Information about national and local support services	*"Locally specific support especially where rehabs are. Maybe a map function that displays 'supports near you'"*<br>*"Would love to have more information on specialised doctors."*
Streamlined navigation	*"I would love there to be a "back" option. I sometimes didn't pay attention to what page I was in and then pressed a button accidentally and struggled to go back to where I was"*<br>*"Simplicity is key - show people where to go after making one choice, then give the option to browse the whole app through the menu navigation button"*
Making the app more interactive	*"Maybe some more interactive activities to complement the quiz"*<br>*"A support feature, i.e. someone you can contact straight away or a support network that is monitored. I.e. somewhere family members, those with a lived experience or those wishing to seek help can chat and support each other."*
Lived experience stories	*"Data base of meth addicts and their journey recovering from meth"*<br>*"Personal stories from ice users and the families"*
Mobile friendly information	*"Less written information, at first look it just looked like pages and pages of written information"*<br>*"It would be useful to have a Frequently Asked Questions section about ice itself (rather than the app). A quick summary of sorts for people who want to skim quickly rather than reading whole sections of text."*<br>*"More visuals"*
